# Complete chloroplast genome of a Chinese endemic species *Corydalis trisecta* Franch. (Papaveraceae)

**DOI:** 10.1080/23802359.2019.1627930

**Published:** 2019-07-11

**Authors:** Nazish Kanwal, Xiao Zhang, Nawal Afzal, Jia Yang, Zhonghu Li, Guifang Zhao

**Affiliations:** Key Laboratory of Resource Biology and Biotechnology in Western China, Ministry of Education, College of Life Sciences, Northwest University, Xi’an, People’s Republic of China

**Keywords:** *Cordalis trisecta* Franch., phylogenetic relationship, chloroplast genome

## Abstract

*Corydalis trisecta* Franch. is an endemic plant found in China. In this study, we presented the first complete chloroplast genome of *C. trisecta,* which was assembled and characterized based on Illumina pair-end sequencing data. The complete chloroplast genome was 161,410 bp in length, with a GC content of 41.4% in total. Its structure contained a large single copy (LSC) region of 89,127 bp and a small single copy (SSC) region of 16,993 bp, which were separated by a pair of extremely inverted repeats (IRs) of 27,645 bp each, with GC content 39.8, 87.2, and 45.2%, respectively. The phylogenetic analysis indicated that *C. triscta* was sister to *Lamprocapons spectabilis* in Papaveraceae.

*Corydalis trisecta* Franch. is a perennial herbaceous plant belonging to the family Papaveraceae included in order Ranunculales. This species is endemic to Qinling Mountain with an altitude of 2500–3300 m in China (Franch, 1894). Recently, it is investigated to be a key species based on field investigation (Wu et al. 2015). However, in spite of its ecological importance, till date, genomic studies have been hindered due to lack of information about the complete chloroplast (cp) genome of *C. trisecta*. In this study, we assembled the cp genome of *C. trisecta* Franch. based on Illumina paired-end sequencing to improve an appreciation of its genomics.

Leaves were collected from a single individual of *C. trisecta* Franch. at the 34°07′24″N, 107°53′31″E, and were dried using silica gel. The genomic DNA was extracted using a modified CTAB protocol (Yang et al. 2014), and genome sequencing was performed by the Illumina Hiseq 2000 Platform (Illumina, San Diego, CA). DNA sample and voucher specimen (No. PHLZH2017105) of *C. trisecta* Franch. were deposited in the Northwest University Museum (NUM). The program NGSQCToolkit_version 2.3.3 was used to trimming all raw reads (Patel and Jain 2012). Then the clean reads were assembled by MIRA version 4.0.2 after dislodging the low quality reads (Chevreux et al. 2004). In total, 1,683,089 bp raw reads were obtained, and cp genome was assembled by MITObim v1.8 (Hahn et al. 2013) with the published sequences of *Coreanomecon hylomeconid* cp genome and *Lamprocapons spectabilis* cp genome as the initial references. The cp genome of *C. trisecta* was annotated using software Geneious v 9.0.2 (Biomatters Ltd., Auckland, NewZealand) by comparison with the cp genome of *C. hylomeconid*. We deposited the annotated cp genome of *C. trisecta* to Genebank with the accession number MK713939.

The whole cp genome was 161,410 bp in length, containing a pair of inverted repeats (IRs) of 27,645 bp each, a large single copy region (LSC, 89,127 bp), and a small single copy region (SSC, 16,993 bp). The cp genome encoded 134 genes, including 85 proteins-coding genes, 37 tRNA genes, and 8 rRNA genes. The nucleotide composition was (28.9% A, 21.0% C, 20.4% G, and 29.7% T) with overall GC content of 41.4%. The GC content in whole cp genome, LSC region, SSC region, and IR region were 38.5, 36.5, 32.6, and 38.5%, respectively.

In order to investigate the phylogenetic status of *C. trisecta*, the available complete cp genomes of 29 species were aligned using MAFFT (Katoh and Standley 2013) with the default parameters. A maximum likelihood (ML) analysis was reconstructed from all of the 29 complete cp genome sequences by RAxML version 7.2.8 (Stamatakis 2006) with 500 bootstrap replicates. The result of a phylogenetic analysis indicated that *C. trisecta* was sister to *L. spectabilis* (Figure 1). Furthermore, the complete cp genome of *C. trisecta* will provide useful genomic information for detailed population genetic studies in the future.

**Figure 1.**
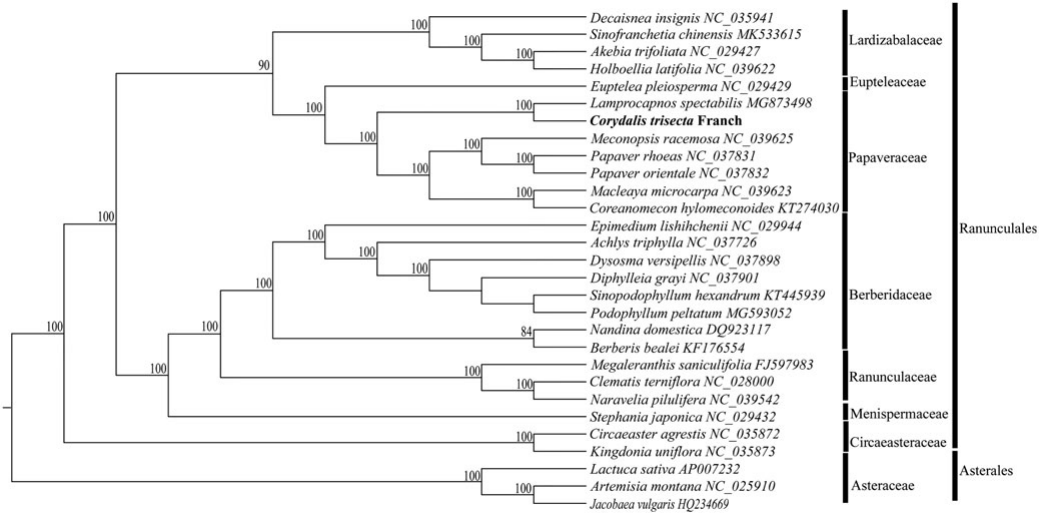
Maximum-likelihood phylogenetic tree based on 29 complete cp genome sequences. The bootstrap values are indicated next to the branches.
